# Genetic and environmental factors associated with alteration of filtration slit proteins and their functions: a scoping review

**DOI:** 10.3389/fneph.2025.1678502

**Published:** 2025-11-24

**Authors:** Aolat Adepeju Adepoju, Mubarak Abubakar Muhammad, Mubashir Mayowa Adamson, Shakirudeen Abdulqodri Adewale, Adedeji Tayyib Adekunle, Lekan Sheriff Ojulari, Abdullateef Isiaka Alagbonsi

**Affiliations:** 1 Department of Physiology, Faculty of Basic Medical Sciences, College of Health Sciences, University of Ilorin, Ilorin, Kwara State, Nigeria; 2 Department of Physiology, School of Medicine and Pharmacy, College of Medicine and Health Sciences, University of Rwanda, Huye, Rwanda

**Keywords:** environment, filtration slit protein, genetics, glomerulus, kidney disease

## Abstract

**Background:**

Filtration slit proteins are important for maintaining the integrity of the glomerular filtration barrier. Genetic mutations and environmental factors can disrupt their structure and functions, leading to proteinuria and kidney diseases. This scoping review aims to synthesize the available information on the genetic and environmental factors that affect the slit proteins to enhance our understanding of the (patho)physiology of glomerular filtration.

**Methods:**

Online databases such as Wiley and PubMed were used. Relevant studies were selected focusing on genetic variations, environmental influences, and their impact on filtration slit proteins. Data extraction and synthesis were conducted to highlight key themes and knowledge gaps.

**Results:**

We summarized at least 20 proteins and their genes, including nephrin, podocin, phospholipase C Epsilon 1 (PLCE1), CD2-Associated Protein (CD2AP), ITGA 3, synaptopodin, myosin 1E (*MYO1E*), flotillin-2 (Flot2), podocalyxin, FAT1, Apo Hemoglobin-Haptoglobin (Apo Hb-Hp), spermidine, P-Cadherin, ephrin B1, Zo- 1 (Zona Occluden), MAGI 1&2 (MAGUK inverted), Par- complex, IP-10 (interferon-inducible protein), neurexin 1, and liver type fatty acid binding protein. We also reported at least 8 environmental factors, including oxidative stress, inflammation, heavy metals, air-bone pollutants, high-fat diets, vitamins and micronutrient deficiency, mechanical stretch, and nephrotoxic agents.

**Conclusion:**

This review highlights various filtration slit proteins and the mechanisms of their alterations by genetic and environmental factors. It contributes to efforts toward personalized therapeutic strategies for disorders of glomerular filtration.

## 1 Introduction

The glomerular filtration barrier, which prevents the passage of substances based on size and charge, consists of three layers: the fenestrated capillary endothelium, a basement membrane composed of type IV collagen and heparan sulfate, and podocyte foot processes (1). These layers contain negatively charged glycoproteins that help restrict the passage of molecules like albumin. The glomerular filtration rate (GFR) measures the amount of fluid filtered from the glomerulus into the Bowman’s capsule per unit of time and is a key indicator of kidney function. The GFR is determined by the Starling equation, which considers hydrostatic and oncotic pressures in the glomerular capillaries and Bowman’s space. Increased hydrostatic pressure in the glomerulus raises GFR, while increased glomerular oncotic pressure or Bowman’s space hydrostatic pressure lowers it, as seen in ureteral constriction (1).

Filtration fraction (FF) represents the proportion of renal plasma flow (RPF) that gets filtered at the glomerulus, typically around 20%. A higher FF increases protein concentration in peritubular capillaries, enhancing reabsorption in the proximal tubule, whereas a lower FF reduces this process (1). The kidneys regulate GFR through autoregulation, maintaining a balance between filtration and reabsorption. The myogenic mechanism responds to increased renal arterial pressure by constricting afferent arterioles, while tubuloglomerular feedback involves the macula densa sensing sodium levels and adjusting arteriole tone and renin release (2). These mechanisms function effectively within an autoregulatory pressure range of 80–180 mm Hg; beyond this range, kidney function can become compromised.

Filtration slit, also known as slit diaphragm or slit pore, is a narrow gap between podocytes containing a large multiprotein complex in the glomerulus of the kidney through which blood is filtered to regulate the passage of molecules. Part of the large multiprotein complex at the filtration slit, nephrin, recruits adaptor proteins to induce signaling to the podocyte cytoskeleton (3, 4). The intricate structure of podocytes allows for ultrafiltration of large volumes of fluid, where small solutes that are necessary for normal clearance of toxic wastes are filtered, while albumin and most other plasma protein components are retained in the bloodstream (5). The alterations in the filtration slit proteins lead to increased glomerular permeability and proteinuria, which is a hallmark of kidney diseases. Several genetic factors associated with the mutation of genes that code for particular proteins have been associated with podocyte injury. Moreover, numerous genetically acquired diseases, including minimal change disease, diabetic nephropathy, focal segmental glomerulosclerosis (FSGS), hypertensive kidney disease, membranous nephropathy, human immunodeficiency virus (HIV)–associated nephropathy, and lupus nephritis, also affect podocytes, causing dysfunction of the filtration barrier (5).

Studies have shown the environmental impacts of various filtration slit protein alterations. Substances such as heavy metals (Cadmium, arsenic, lead, mercury), which are products of environmental pollution and industrial activities, have been reported to be nephrotoxic. Other substances, such as Polystyrene microplastics (PSMPs), phthalates, and bisphenol A, which are commonly used as plasticizers, have been documented to have strong nephrotoxic effects. Some antibiotics, like puromycin, a known aminoglycoside that inhibits protein synthesis, have been proven to cause injury to podocytes. Furthermore, some viral infections, such as HIV and COVID-19, have been linked to causing protein loss in the filtration slit.

Recently, we synthesized available evidence on the factors associated with aminoacidurias, highlighting 9 genes and some environmental factors (6). While many studies have been done about factors that affect the proteins in the filtration slit, the available information is scattered, making it difficult for educators, researchers, and clinicians to have a full grasp of the information. Thus, this review presents the genetic and environmental factors that cause alteration of the filtration slit proteins while briefly explaining their manifestations on kidney function. It is believed that the information will enhance our understanding of the (patho)physiology of glomerular filtration.

## 2 Methodology

### 2.1 Literature search

This scoping review adhered to the Preferred Reporting Items for Systematic reviews and Meta-Analyses-extension for Scoping Reviews (PRISMA-ScR) checklist as outlined by Tricco et al. (7*)* The review framework followed the six stages of scoping review (8) including: 1) formulating the research questions (**Table 1**), 2) identifying relevant literature, 3) selecting applicable studies, 4) mapping the data, 5) summarizing and synthesizing the findings, and 6) consulting experts. An electronic literature search was conducted primarily through the Wiley Online Library and PubMed to find peer-reviewed articles. The review focused on studies conducted across various continents: Europe, the Americas, Asia, and Africa. The search strategy employed a combination of controlled vocabulary and free-text terms, including phrases such as “slit diaphragm proteins”, “nephrin”, “podocin”, “P-cadherin”, along with “genetic alterations”, “environmental stressors”, “oxidative injury”, and related terms. Boolean operators (AND/OR) were applied to optimize search sensitivity and specificity. The search, conducted in April 2025, was confined to literature published between January 1995 and 31^st^ March 2025, restricted to studies available in the English language, and focusing on both human and relevant mammalian animal models. The search strings for each database can be found in the **Supplementary File S1**.

**Table 1:** PICO elements that guided the research questions of the review.

PICO element	Inclusion criteria
Population (P)	Human and mammalian animal models
Intervention (I)	Genetic and environmental factors contributing to the alteration of filtration slit proteins.
Comparison (C)	Comparison between genetic and environmental factors that contribute to the alteration of filtration slit proteins
Outcome (O)	Glomerular diseases

### 2.2 Selection criteria

To qualify for inclusion, studies needed to address either genetic mutations or external environmental influences affecting the expression, localization, or function of one or more key slit diaphragm proteins. Eligible study designs included laboratory experiments, clinical research, and observational studies with mechanistic insights. Excluded materials encompassed narrative reviews, commentaries, conference summaries, and works not directly engaging with slit diaphragm biology. A data extraction tool was constructed to record essential study characteristics, including authorship, publication date, study setting, methodology, proteins of interest, implicated genetic or environmental factors, and major outcomes (**Supplementary File S2**). The extracted data were organized thematically, with emphasis placed on two broad categories: (i) Genetic determinants influencing the slit diaphragm architecture; and (ii) Environmental factors contributing to pathological alterations in protein structure or function. The evidence was summarized narratively, and major research trends and gaps were identified to guide future investigations. The screening process was performed independently by two reviewers, with discrepancies resolved through consensus or by a third reviewer.

## 3 Results

### 3.1 Selected articles

**Figure 1:**
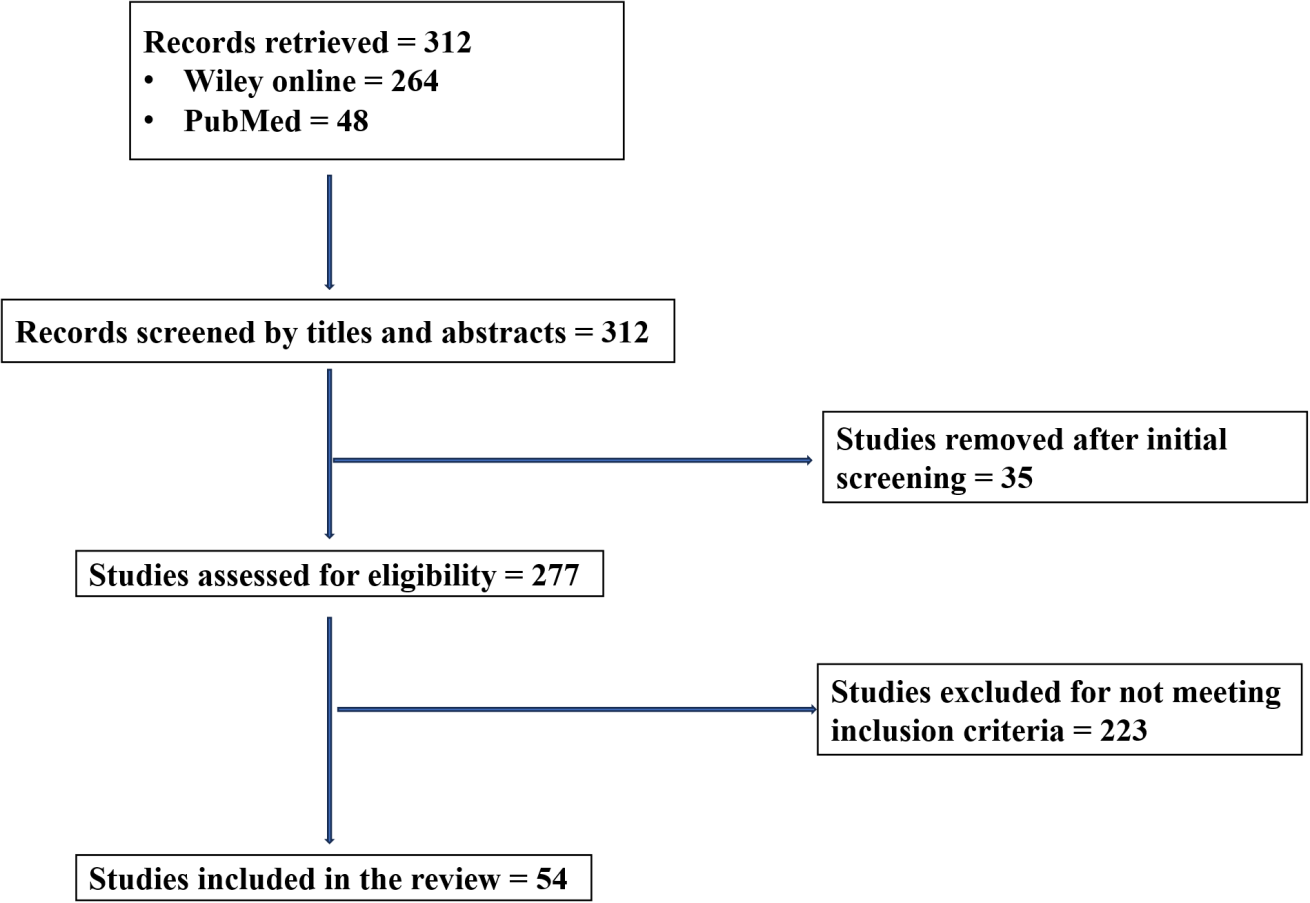
PRISMA flow chart of the study selection process.

An initial pool of 312 publications was identified (i.e., 264 from Wiley and 48 from PubMed) and screened for their titles and abstracts. After removing duplicates and irrelevant articles during the initial screening (n = 35) and non-eligible articles based on the inclusion criteria (n = 223), 54 studies fulfilled all the predefined criteria and were incorporated into the final synthesis (**Figure 1**).

### 3.2 Filtration slit proteins

The number of known filtration slit proteins in humans is still expanding as research advances. Currently, at least 10–15 well-characterized proteins are essential for slit diaphragm integrity, while over 20 additional proteins have been identified as potential regulators or associated components. Some of these proteins include nephrin, podocin, phospholipase C Epsilon 1 (PLCE1); CD2-Associated Protein (CD2AP); ITGA 3, synaptopodin; NEPH-1, myosin 1E (*MYO1E*), flotillin-2 (Flot2), podocalyxin, FAT1, Apo Hemoglobin-Haptoglobin (Apo Hb-Hp), spermidine, P-Cadherin, ephrin B1, Zo- 1 (Zona Occluden), MAGI 1&2 (MAGUK inverted), Par- complex, IP-10 (interferon-inducible protein), neurexin 1, and liver type fatty acid binding protein. Herein, we present some of the genetic (**Table 2**) and environmental (**Table 3**) factors that are associated with the alteration in the structure and functions of these proteins.

**Table 2:** Genetic factors associated with alteration in filtration slits protein.

S/No	Slit proteins	Genes encoding the protein	Genetic methods used in identifying the gene	Nature of the molecular presentation of the gene in glomerular disease	Authors
1	Nephrin	NPHS1	Positional cloning	Mutation	Putaala et al. (9*)*
2	Podocin	NPHS2	Positional cloning	Mutation	Huber et al. (5)
3	CD2 Associated Protein	CD2AP	Immunoblotting	Mutation	Yu et al. (10*)*
4	Phospholipase C Epsilon 1	PLCE1	Positional cloning	Mutation	Hinkes et al. (11*)*
5	Integrin α3	ITGA3	Polymerase Chain Reaction (PCR)	Mutation	Has et al. (12*)*
6	Synaptopodin	SYNPO	Immunoblotting	Mutation, Polymorphism	Mundel et al. (13*)*
7	Myosin 1E	MYO1E	High-Throughput Sequencing (HTS)/Immunohistochemistry	Mutation	Mele et al. (14*)*
8	Flotillin	FLOT2	Polymerase Chain Reaction (PCR)	Mutation	Yu et al. (10*)*
9	Podocalyxin	PODXL	EXOME Sequencing	Mutation	Barua et al. (15*)*
10	P-cadherin	CDH3	Polymerase Chain Reaction (PCR)	Mutation	Reiser et al. (16*)*
11	FAT	FAT1	Reverse transcriptase Polymerase Chain Reaction (RT-PCR)	Mutation	Yaoita et al. (17*)*
12	ApoHemoglobin-Haptoglobin	ApoHb-Hp	Polymerase Chain Reaction (PCR) and Electrophoresis	Mutation, Polymorphism	Lucas et al. (18*)*
13	Spermidine	N/A	Immunohistochemistry	Mutation	Zhang et al. (19*)*
14	Nephrin-Binding Ephrin	Ephrin-B1	Reverse transcriptase Polymerase Chain Reaction (RT-PCR) and immunohistochemical	Dysmorphism	Fukusumi et al. (20*)*
15	Membrane-associated guanylate kinase inverted 2	MAGI 2	EXOME Sequencing	Mutation	Bierzynska et al. (21*)*
16	Par-complex	Par3-Par6-aPKC	*In-situ* hybridization, Real time Polymerase Chain Reaction (RT PCR)	Mutation	Hartleben et al. (22*)* Takamura et al. (23*)*
17	IP-complex	IQGAP1	Immunohistochemistry	Mutation	Rigothier et al. (24*)*
18	IP-10	CXCL10	Reverse Transcriptase Polymerase Chain Reaction (RT-PCR)	Mutation	Matz et al. (25*)*
19	Neurexin-1	NRXN1	Immunohistochemistry	Mutation	Saito et al. (26*)*
20	Liver fatty acid binding protein Z	L-FABP	Immunohistochemistry	Mutation	Xu et al. (27*)* Mitsides et al. (28*)*

**Table 3:** Environmental factors associated with alteration in filtration slit proteins.

S/No	Environmental factor	Affected slit proteins	Environmental tool used for identification	Authors
1.	Oxidative stress	Nephrin, Synaptopodin, Podocin	Immunohistochemistry, electron microscope	Wang et al. (2*)*
2.	Inflammation	Podocin, CD2AP, Synaptopodin, MAGI2, FAT1, Spermidine	ELISA for TNF-α, IL-6; Renal Biopsy, Western Blot	Luo et al. (29*)*
3.	Heavy metals	Podocin, Nephrin	Atomic Absorption Spectroscopy (AAS), Western Blot	Hartleben et al. (22*)*
4.	Air-bone disease	Podocin, CD2AP	Air Quality Monitoring, Lung Function Tests, Immunohistochemistry	Russo et al. (30*)*
5.	High-fat diet	Podocin, Nephrin	Dietary Recall, Blood Lipid Profiling, Western Blot	Pan et al. (31*)*
6.	Vitamins and micronutrients deficiency	Podocin, Nephrin	Serum 25(OH)D Measurement, Immunohistochemistry	Saleem et al. (32*)*
7.	Mechanical stress and stretch	ITGA3, FAT1, podocalyxin, MAGI2, and ZO-1	Cyclic stretch receptor	Endlich et al. (33*)*
8.	Nephrotoxic agents	SYNPO, PLCE1, *MYO1E*, FLOT2, NPHS2 and CD2AP	Immunohistochemistry, immunofluorescence microscopy, and Western blotting.	Li et al. (34*)*

#### 3.2.1 Genetic factors

##### 3.2.1.1 Nephrin

Nephrin, encoded by *NPHS1*, is a crucial protein located at the slit diaphragm of glomerular podocytes, playing an essential role in the glomerular filtration barrier (35). It is a transmembrane protein whose extracellular component forms the core of the slit diaphragm (36). The *NPHS1* gene is located on chromosome 19q13.1 with 29 exons and a size of 26 kb. Mutations in *NPHS1* can lead to congenital nephrotic syndrome, characterized by proteinuria and absence of slit diaphragms (9). Free cysteine is present in the extracellular domain of the nephron, which allows disulfide bonds to be formed with adjacent molecules. The stability and maintenance of slit diaphragm’s health require *Cis* and *trans* homophilic and heterophilic interactions of nephrin with itself and with Neph family proteins (Neph1, Neph2, and Neph3) (22). Even at the first stage of almost all types of proteinuric diseases, when no changes of other podocyte proteins, such as podocin, can be detected, Nephrin alteration is noticeable (37).

Nephrin functions not only as a structural component but also as a signaling scaffold, influencing podocyte adhesion, shape, and survival (38). Its function has been further elucidated by the absence of this protein in birds, making them have larger slit diaphragms (37). Its role in the development of cardiac vessels has recently been highlighted too (39). It also functions as a synaptic adhesion molecule, as its orthologues in Drosophila melanogaster (Hibris) are crucial players in synapse targeting and positioning (40). Studies have shown that decreased nephrin expression is associated with various proteinuric kidney diseases. In adult mice, induced nephrin deletion leads to progressive proteinuria and FSGS, despite initial normal foot process ultrastructure. Furthermore, podocytes with low nephrin expression are more susceptible to injury and unable to recover following perturbation (4). Therefore, decreased nephrin expression may contribute to disease progression independently of podocyte loss.

##### 3.2.1.2 Podocin

Podocin, another important protein, interacts with nephrin and is essential for the proper functioning of the slit diaphragm (4). Podocin is encoded by the *NPHS2* gene (*Nephrosis 2, Idiopathic, Steroid-Resistant*), located on chromosome 1q25–q31. *NPHS2* mutations are associated with autosomal recessive steroid-resistant nephrotic syndrome (SRNS). Over 30 pathogenic mutations have been identified, affecting podocin’s function or localization (6). Although *NPHS2* is the primary gene coding for podocin, its interaction network includes *NPHS1* (Nephrin), CD2AP, TRPC6, and WT1 (Wilms Tumor 1). Thus, at least 4–5 key genes influence podocin’s function and regulation, forming a complex signaling and structural unit at the slit diaphragm (7). Immunofluorescence and confocal microscopy have been used to visualize podocin at the slit diaphragm, while Next-Generation Sequencing (NGS) and Sanger Sequencing have been used to study mutations in *NPHS2*.

##### 3.2.1.3 Phospholipase C Epsilon 1

The PLCE1 is a signaling enzyme that plays a crucial role in podocyte function and cytoskeletal organization. It regulates intracellular pathways involved in filtration slit stability. PLCE1 is encoded by the *PLCE1* gene, located on chromosome 10q23. It encodes a large protein (more than 2,000 amino acids) with multiple functional domains, including: Ras-associating (RA) domains, CDC25-like GEF domain, and Catalytic X and Y domains for phospholipase activity. Mutations in *PLCE1* cause autosomal recessive nephrotic syndrome type 3 (NPHS3), with variable onset and responsiveness to steroids (11). Its mutations can also cause congenital and childhood-onset nephrotic syndrome, particularly diffuse mesangial sclerosis (DMS) and FSGS (11). Biallelic loss-of-function mutations in *PLCE1* disrupt the phosphatidylinositol signaling pathway, leading to defective podocyte cytoskeleton organization and increased susceptibility to podocyte injury (41). Certain missense mutations result in partial loss of *PLCE1* function, which may not cause immediate disease but can contribute to progressive glomerular damage in response to additional stressors (42). These mutations impair the activation of Ras-related GTPases and protein kinase C (PKC), leading to podocyte detachment and proteinuria (43). Some heterozygous *PLCE1* variants have been associated with an increased risk of nephrotic syndrome, indicating that gene-environment interactions may play a role in disease onset (5). *PLCE1* interacts with several genes and proteins important in podocyte signaling and glomerular filtration. For instance, *NPHS1* and *NPHS2* interact indirectly with it via shared signaling pathways. Also, WT1 serves as a transcription factor that regulates *PLCE1* expression in podocytes, while *ACTN4* (Alpha-actinin-4) and ARHGAP24 are cytoskeletal genes that work in the pathways influenced by *PLCE1*. Thus, 4–6 genes are known to functionally interact or co-regulate with *PLCE1* in podocytes (5, 44). Sanger sequencing and NGS panels have been used to identify *PLCE1* mutations in nephrotic syndrome patients, while RT-qPCR has also been used for measuring *PLCE1* mRNA expression in renal tissues.

##### 3.2.1.4 CD2-associated protein

CD2-associated protein (*CD2AP*) is a scaffolding protein that plays a critical role in podocyte cytoskeletal organization, actin remodeling, and intracellular signaling. It interacts with nephrin and podocin to maintain slit diaphragm integrity and regulate endocytosis (45). Given its role in podocyte function, genetic variations and environmental stressors affecting CD2AP expression contribute to proteinuric kidney diseases, particularly FSGS (46).

CD2AP is encoded by the *CD2AP* gene, located on chromosome 6p12. It encodes a multidomain protein containing three SH3 domains, a coiled-coil region, and a proline-rich domain. CD2AP is essential for nephrin trafficking, cytoskeletal dynamics, and podocyte survival. Mutations or haploinsufficiency of CD2AP have been linked to FSGS and Alzheimer’s disease (AD) (10, 46). Homozygous mutations in the *CD2AP* gene lead to early-onset nephrotic syndrome, characterized by severe proteinuria, podocyte effacement, and rapid progression to end-stage renal disease (ESRD) (46). Even heterozygous *CD2AP* mutations have been identified as a risk factor for adult-onset FSGS, suggesting a dosage-sensitive role in maintaining podocyte health. Research on *CD2AP*-deficient mice has shown that a 50% reduction in *CD2AP* expression is sufficient to cause glomerular injury, proteinuria, and podocyte apoptosis (47). This supports the idea that CD2AP haploinsufficiency weakens the actin cytoskeleton, making podocytes more susceptible to mechanical stress and injury (48). Interestingly, genetic variants of *CD2AP* have been linked to AD, suggesting a shared role in cytoskeletal regulation between neurons and podocytes (46). This highlights CD2AP’s broader importance in cellular function beyond the kidney.

CD2AP interacts with a molecular network of 4–6 major proteins/genes at the slit diaphragm. NPHS1 binds CD2AP to mediate actin linkage, NPHS2 co-regulates nephrin–CD2AP interactions, FYN is a Src-family kinase involved in CD2AP phosphorylation, ACTN4 links to cytoskeletal regulation, while TRPC6 is a calcium channel modulated by CD2AP-nephrin complex. These interactions position CD2AP as a central node in podocyte structure and signaling (47). Western Blotting is used to detect CD2AP protein levels in tissues, qRT-PCR for quantifying CD2AP mRNA expression, and Immunoprecipitation & Co-IP to study protein–protein interactions with nephrin/podocin.

##### 3.2.1.5 ITGA3

The ITGA3, localized to the basal surface of podocytes, interacts with the glomerular basement membrane (GBM) components like laminin-521 (α5β2γ1) to maintain filtration barrier integrity. Dysregulation of ITGA3 can lead to podocyte dysfunction, proteinuria, and kidney disease (49). About 4–6 genes are functionally associated with ITGA3 in the context of podocyte biology and GBM interaction. ITGB1 (Integrin β1) forms a dimer with ITGA3 for laminin binding, LAMA5 (Laminin α5) forms the primary ligand in the GBM for α3β1 integrin, CD151 interacts with a tetraspanin protein that stabilizes α3β1 integrin complexes, NPHS1 & NPHS2 are indirectly associated via cytoskeletal interactions and slit diaphragm regulation, while COL4A3/4/5 interacts with GBM collagen chains with which integrins indirectly interact (50). Biallelic mutations in *ITGA3* are linked to congenital nephrotic syndrome, interstitial lung disease, and epidermolysis bullosa. These mutations disrupt α3β1 integrin function, leading to defective cell-matrix interactions (12). Single-nucleotide polymorphisms (SNPs) in ITGA3 may influence susceptibility to kidney diseases like FSGS or diabetic nephropathy, though research is ongoing (51). Sanger sequencing and WES have been used to identify mutations of ITGA3 in patients with nephrotic syndrome or ILNEB syndrome. Immunohistochemistry and immunofluorescence for spatial localization of ITGA3 in kidney tissues, qRT-PCR and Western blotting to quantify mRNA and protein levels, and electron microscopy to visualize GBM abnormalities and podocyte foot process effacement have been documented (51).

##### 3.2.1.6 Synaptopodin

Synaptopodin is highly expressed in podocytes, where it interacts with actin filaments and other slit diaphragm proteins, such as nephrin and CD2AP. It stabilizes the podocyte foot processes and ensures the proper functioning of the glomerular filtration barrier. Loss or dysfunction of synaptopodin disrupts the slit diaphragm, leading to proteinuria and progressive kidney damage (48, 52). Dysregulation of synaptopodin is linked to proteinuric kidney diseases, such as FSGS and diabetic nephropathy. Variations in the synaptopodin gene (*SYNPO*) have been associated with susceptibility to kidney diseases. For example, SNPs in *SYNPO* may influence protein expression levels or function, contributing to glomerular diseases (53). DNA methylation and histone modifications can regulate synaptopodin expression, and thus, hypermethylation of the *SYNPO* promoter has been observed in diabetic nephropathy, leading to reduced synaptopodin levels and podocyte injury (54). Synaptopodin interacts with genes encoding other slit diaphragm proteins, such as *NPHS1* and *NPHS2*. Thus, mutations in these genes can indirectly affect synaptopodin’s function and stability (55).

##### 3.2.1.7 Myosin 1E

Myosin 1E (coded by the *MYO1E* gene) is a member of the myosin superfamily, which is involved in various cellular processes, including cytoskeletal organization, membrane dynamics, and intracellular transport. Myosin IE is particularly significant in the kidney, where it plays a crucial role in maintaining the integrity of the glomerular filtration barrier, specifically at the filtration slit diaphragm of podocytes. Mutations or dysregulation of *MYO1E* can lead to kidney diseases, such as autosomal recessive steroid-resistant nephrotic syndrome (SRNS), which disrupts the protein’s function, leading to podocyte injury and proteinuria. A study identified homozygous mutations in *MYO1E* in patients with SRNS (14), highlighting its role in maintaining the glomerular filtration barrier (12). The SNPs in *MYO1E* may influence its expression or function, potentially contributing to susceptibility to kidney diseases. Also, epigenetic changes, such as DNA methylation or histone modifications, may regulate *MYO1E* expression, and the dysregulation of these processes could impair podocyte function (56). About 5–6 genes/proteins are involved in *MYO1E*’s filtration slit network: *ACTN4* is responsible for the cytoskeletal actin-bundling protein, *CD2AP* is for a scaffold protein that links slit diaphragm to actin, synaptopodin regulates actin dynamics and stabilizes *MYO1E*. Furthermore, *NPHS1* and *NPHS2* are indirectly influenced by cytoskeletal organization and dynamin 2 partners in endocytosis and membrane remodeling (55).

##### 3.2.1.8 Flotillin-2

In goldfish, upregulation of the Flotillin-2 gene (*Flot2*) during axon regeneration and optic nerve lesions was initially identified. Subsequently, the crucial role of *Flot2* in neural differentiation was established (10, 46). Furthermore, its formation of homo- and hetero-oligomers as a scaffold for lipid rafts and interaction with cytoskeletal proteins like actin and tubulin via their stomatin/prohibitin/flotillin/HflK/C(SPFH) domains has been reported (10). In the nephron, the vital role of *Flot2* is to attenuate podocyte injury by recruiting the podocin-nephrin complex into rafts (10). The *Flot2* gene is co-expressed with *FLOT1*, and both proteins form hetero-oligomeric complexes at the plasma membrane. While mutations in *Flot2* have not been directly implicated in monogenic nephrotic syndromes, altered expression of *Flot2* has been reported in kidney diseases such as FSGS and diabetic nephropathy (10). These changes may reflect a compensatory genetic response to podocyte injury. In disease models characterized by foot process effacement, increased *Flot2* expression correlates with the disruption of slit diaphragm integrity, suggesting a genetic regulatory mechanism attempting to stabilize damaged membrane domains (10). Flotillin-2 functionally interacts with *Flot1* in organizing lipid raft domains, CD2AP, and podocin, likely through scaffold-mediated clustering within raft regions. Genes regulating the Rho family of GTPases (e.g., RAC1, CDC42) affect cytoskeletal remodeling and podocyte motility. Altered activity or expression of these related genes can affect *Flot2* localization and function, leading to filtration barrier dysfunction. Methodologically, the CRISPR/Cas9-mediated knockout technique was used in a Chinese study where *Flot2* expression was significantly altered in mouse models of renal fibrosis and glomerular injury (34). Also, siRNA-mediated gene silencing was used in a German study where *Flot2* depletion disrupts podocyte shape and actin structure. Lastly, RNA sequencing (RNA-seq) has been used to compare *Flot2* expression levels in diseased versus normal glomeruli (34).

##### 3.2.1.9 Podocalyxin

In the podocyte, podocalyxin (*PCX*) forms a meshwork to support the capillaries at the apical surface and also contributes to other structures in the filtration slit. It is also expressed in the hematopoietic progenitor cells, neurons, and vascular epithelium as a transmembrane O-glycosylated and sialylated protein (15, 16). It is secreted in the urine following damage to the podocyte that results from some kidney diseases. Thus, it is useful as a clinical measure of the extent of glomerular damage in various kidney diseases (15).

##### 3.2.1.10 P-Cadherin

P-Cadherin is a member of the cadherin family of cell surface glycoproteins that mediate Ca^2+^-dependent cell-cell adhesion and is expressed differentially in normal epithelial tissues (57). It was detected at the slit diaphragm in association with Zona Occludens-1 with α, β, & γ-catenin in 1999 (16). The *CDH3* gene, located on chromosome 16q22.1, encodes the P-cadherin protein, a calcium-dependent cell-cell adhesion glycoprotein. This protein plays a crucial role in maintaining epithelial integrity and is involved in various developmental processes.

##### 3.2.1.11 FAT1

FAT1 is a member of a small family of vertebrate cadherin-like genes, designated FAT1–FAT4 in humans, whose orthologues were first recognized in Drosophila (58). The FAT1 protein contains 33 cadherin repeats, followed by 5 epidermal growth factor (EGF)-like repeat domains, a laminin G domain, a transmembrane domain, and an intracellular domain. FAT1 is a large gene located on chromosome 4q35.2 and encodes a protein involved in maintaining podocyte structure and intercellular adhesion. Disruption of FAT1 function can compromise the slit diaphragm, a specialized intercellular junction in podocytes, leading to proteinuria and progressive kidney disease. FAT cadherins play a role in cell migration, lamellipodia dynamics, cell polarity, and cell–cell adhesion. Fat cadherins have been reported to interact with Ena/VASP proteins, atrophins, β-catenin, scribble, and HOMER1–HOMER3, thereby influencing Wnt and Hippo signaling and the regulation of planar cell polarity (PCP), the process by which cells become polarized and organized within the plane of an epithelial sheet. The Fat1^-/-^ mouse displays abnormal podocyte foot processes, brain developmental defects, and eye abnormalities (58). A study showed that a FAT1 defect can lead to a reduction in cell migration and cell–cell adhesion, FSGS due to podocyte-specific FAT1 loss-of-function, and CDC42-mediated renal tubular defects due to FAT1 loss. FAT1 interacts with several key slit diaphragm-associated proteins and signaling cascades, including nephrin, podocin, CD2AP, and TRPC6, essential for glomerular homeostasis (59). Mutations in the *FAT1* gene have been increasingly recognized as contributors to both isolated and syndromic forms of glomerular disease. Epigenetic modifications, including promoter methylation or histone deacetylation, are potential regulatory mechanisms for FAT1 expression in podocytes, although direct evidence is still emerging (17).

##### 3.2.1.12 ApoHemoglobin-Haptoglobin

ApoHemoglobin–Haptoglobin (ApoHb–Hp) is a synthetic chimeric protein designed to stabilize and scavenge free hemoglobin and heme in conditions of intravascular hemolysis. The haptoglobin (Hp) portion of the complex is encoded by the HP gene located on chromosome 16q22. The hemoglobin (Hb) subunits derive from the HBA1, HBA2 (alpha globins), and HBB (beta globin) genes, located on chromosomes 16 and 11, respectively. The primary function of this engineered protein complex is to prevent hemoglobin-induced oxidative stress by binding and stabilizing free hemoglobin and heme. This protects renal tissues from damage during episodes of hemolysis and facilitates safe clearance through the liver (60).

A study conducted in Switzerland (42) identified the ApoHb–Hp complex using recombinant protein engineering, co-immunoprecipitation, spectrophotometry, and Western blot analysis to confirm the binding interactions between the Hp domain and hemoglobin dimers, as well as the heme-binding capacity of ApoHb. Similarly, studies have used protein expression assays, heme-binding affinity measurements, and *in vivo* murine models to validate the dual scavenging properties of the ApoHb–Hp complex, emphasizing its potential therapeutic application in conditions such as sickle cell disease and hemolytic uremic syndrome (41, 61).

##### 3.2.1.13 Spermidine

Spermidine, a polyamine with known cytoprotective properties, plays a significant role in maintaining glomerular homeostasis. In the glomerulus, spermidine activates cell death in podocytes and other glomerular cells, a process critical for removing damaged cellular components and maintaining filtration barrier integrity. This action is mediated through the inhibition of acetyltransferase EP300 (E1A-associated protein p300), leading to reduced acetylation of autophagy-related proteins (ATG proteins) and enhanced autophagic flux (62). The biosynthesis and regulation of spermidine involve several genes like ODC1 (Ornithine decarboxylase 1), which catalyzes the conversion of ornithine to putrescine (a spermidine precursor); SRM (Spermidine synthase), which converts putrescine to spermidine; and AMD1 (Adenosylmethionine decarboxylase 1), which provides the aminopropyl group for this conversion.

Using immunofluorescence microscopy, the spatial localization of filtration slit proteins in murine glomeruli was visualized in China following spermidine treatment (63). Their study confirmed that spermidine enhances the localization and stability of podocyte slit diaphragm components, such as nephrin and podocin, likely via autophagy-mediated preservation of podocyte structure.

##### 3.2.1.14 Sphrin B1

Sphrin B1, encoded by the *EPHB2* gene, is a member of the ephrin receptor-interacting protein family and is a component of the filtration slit. Sphrin B1 plays a key role in maintaining podocyte structure and regulating cytoskeletal dynamics essential for glomerular filtration. Sphrin-B1 was expressed at the slit diaphragm and interacted with nephrin, a key molecule of the slit diaphragm (64). The *EPHB2* gene is located on chromosome 1p36.12 and encodes a transmembrane protein that facilitates cell-cell adhesion and repulsion signaling. Using immunofluorescence (IF), it was found that Sphrin-B1 was expressed along the glomerular capillary loop. Immunoelectron microscopy revealed that Sphrin-B1 expression was restricted to the slit diaphragm (65).

Sphrin B1 forms cis- and trans-interactions with nephrin and other slit diaphragm-associated proteins, contributing to the stability and flexibility of the filtration barrier (66). Mutations or reduced expression of Sphrin B1 have been implicated in cytoskeletal disorganization, foot process effacement, and proteinuria (64). Genetic studies demonstrate that knockdown of Sphrin B1 in animal models leads to impaired podocyte architecture and glomerular injury (66). Apart from its structural role, Sphrin B1 is involved in intracellular signaling that governs podocyte survival, actin cytoskeleton rearrangement, and cell polarity. It activates downstream effectors such as Rho family GTPases, which are essential for the maintenance of foot process integrity (66). Additionally, genetic alterations affecting Sphrin B1 signaling may increase susceptibility to podocyte detachment and glomerulosclerosis, independent of nephrin expression levels (64). Experimental evidence has further highlighted the cooperative role of Sphrin B1 with other slit diaphragm proteins in responding to mechanical stress and inflammatory insults, making it a key player in maintaining long-term filtration slit stability.

##### 3.2.1.15 Zona occludens 1

Zonula Occludens 1 (ZO-1), encoded by the *TJP1* gene, belongs to the uridine kinase family. It was the first tight junction protein to be identified, and contains the PDZ, SH3, and uridine acid regions. ZO-1 is mainly expressed on the cytoplasmic side of the foot process of glomerular podocytes near the slit diaphragm and links the slit proteins through its PDZ domain to the actin cytoskeleton (67). It is a tight junction protein that plays an essential role in maintaining the integrity of the podocyte filtration barrier (63). Therefore, the correct localization and expression of ZO-1 in the tight junction between the podocytes are particularly important for the maintenance of podocyte permeability. Previous studies have demonstrated that podocyte-specific depletion of ZO-1 leads to damage to slit diaphragm integrity, thus causing proteinuria (63). Mutations in the TJP1 gene or dysregulation of its expression have been associated with the destabilization of the actin cytoskeleton in podocytes, increased permeability of the glomerular filtration barrier, and progression of proteinuric kidney diseases. Besides, ZO-1 can not only regulate the stability of the slit protein nephrin, but can also regulate cytoskeleton organization by binding to F-actin (68). Therefore, clarification of the regulation mechanism of ZO-1 holds considerable clinical importance.

##### 3.2.1.16 MAGI 1 and 2

MAGI-1 and MAGI-2 (Membrane-Associated Guanylate Kinase Inverted-1 and 2) are important in organizing protein complexes at the filtration slit of glomerular podocytes. These proteins are encoded by the *MAGI1* and *MAGI2* genes, located on chromosomes 3p14.1 and 7q21.11, respectively. They belong to the membrane-associated guanylate kinase (MAGUK) family and are essential for the stability and signaling of the slit diaphragm (69).

Diminished MAGI-1 expression in cultured podocytes reduced nephrin and *NEPH1* membrane localization and weakened tight junction integrity (70). In cultured podocytes, MAGI-1 depletion reduced intercellular contact-induced Rap1 activation, a pathway critical for proper podocyte function. Similarly, MAGI-1 knock-out mice showed diminished glomerular Rap1 activation, an effect dramatically enhanced by concomitant nephrin haploinsufficiency. Combined overexpression of MAGI-1 and nephrin increased Rap1 activation, but not when substituting a mutant MAGI-1 that cannot bind nephrin. The authors concluded that the interaction between nephrin and MAGI-1 regulates Rap1 activation in podocytes to maintain long-term slit diaphragm structure (70).

MAGI-2 downregulation coincided with a reduced expression of slit-diaphragm backbone proteins in human glomerular disease, such as FSGS or IgA nephropathy. Podocyte-specific deficiency of MAGI-2 in mice abrogated the localization of Nephrin and Neph1 independently of other scaffold proteins. Although a deficiency of zonula occludens-1 downregulated the endogenous Neph1 expression, MAGI-2 recovered Neph1 expression at the cellular edge in cultured podocytes (69).

##### 3.2.1.17 Par-complex

The Par complex (comprising Par3, Par6, and aPKC) governs cell polarity in podocytes. It localizes to the slit diaphragm and orchestrates the asymmetric distribution of membrane proteins and cytoskeletal components. The complex ensures directional vesicle trafficking and maintenance of podocyte architecture under mechanical stress (43, 71).

##### 3.2.1.18 IP-10

IP-10 (*CXCL10*) is a chemokine that influences podocyte biology indirectly. In glomerular diseases, elevated IP-10 promotes Th1 cell recruitment, inflammatory cytokine release, and podocyte injury, leading to altered expression of filtration slit diaphragm proteins such as nephrin and podocin. IP-10 levels correlate with inflammatory glomerular injury severity and can act as a biomarker of ongoing immune-mediated damage. Also, in diabetic nephropathy and lupus nephritis, IP-10 elevation contributes to podocyte effacement and loss of slit diaphragm integrity, exacerbating proteinuria (72).

##### 3.2.1.19 Neurexin 1

Neurexin 1 is primarily a synaptic adhesion molecule; neurexin-neuroligin-like interactions have been identified in podocyte slit diaphragm complexes. Neurexin 1 expression in podocytes suggests potential roles in maintaining slit diaphragm structural integrity, similar to synaptic adhesion in neurons. It may act as a structural stabilizer or signaling modulator within the filtration barrier, though its direct role in glomerular pathology remains under investigation.

Alterations in Neurexin 1 expression could disrupt podocyte adhesion and slit diaphragm function, contributing to proteinuria (26).

##### 3.2.1.20 Liver fatty acid binding protein

Liver fatty acid binding protein is expressed in proximal tubules but has an indirect role in glomerular health. Elevated urinary liver fatty acid binding protein indicates proximal tubular stress secondary to glomerular filtration barrier damage. Podocyte injury with loss of slit diaphragm proteins like nephrin increases filtered protein load, burdening tubules and elevating liver fatty acid binding protein secretion. It is a sensitive biomarker for early tubular injury in proteinuric states, reflecting upstream glomerular filtration slit dysfunction. Also, in diabetic nephropathy, glomerular slit diaphragm disruption leads to albuminuria, tubular reabsorption stress, oxidative injury, and upregulation of *L-FABP* (73).

#### 3.2.2 Environmental factors

##### 3.2.2.1 Oxidative stress

Oxidative stress is a major environmental factor that contributes to the malfunction of filtration slit proteins in podocytes. Increased oxidative stress, often associated with hyperglycemia, hypertension, and toxin exposure, can downregulate nephrin expression and promote podocyte apoptosis. Nephrin, as a key structural protein of the slit diaphragm, has been identified as a target of autoantibodies in these conditions. Oxidative stress disrupts almost all filtration slit proteins’ structural integrity, leading to impaired slit diaphragm function. Oxidative stress has also been shown to negatively affect endogenous spermidine levels and disrupt polyamine metabolism in renal tissues. A study in the United States reported that elevated reactive oxygen species (ROS) downregulate polyamine biosynthetic enzymes, including ODC1 and SRM, thereby reducing spermidine levels in the kidney (74). Circulating anti-nephrin autoantibodies appear to correlate with disease activity in Minimal Change Disease (MCD) and Idiopathic Nephrotic Syndrome (INS), suggesting their involvement in disease pathogenesis rather than being mere byproducts of podocyte injury. Their binding at the slit diaphragm disrupts podocyte integrity, impairing filtration barrier function and contributing to proteinuria. Also, ROS can downregulate podocin expression, CD2AP, and Flot2 and disrupt their localization (75). These findings underscore their relevance as both biomarkers and potential therapeutic targets.

##### 3.2.2.2 Inflammation

Pro-inflammatory cytokines such as TNF-α, IL-1β, and IL-6 alter the expression, localization, and modifications of many slit diaphragm proteins like nephrin, podocin, ITGA3, CD2AP, and ZO-1. A study conducted in Germany by (76) demonstrated that inflammatory cytokines reduce ODC1 transcription and impair spermidine production in renal epithelial and glomerular cells. Using cytokine assays, chromatin immunoprecipitation, and Western blotting, the study showed that inflammatory signaling disrupts the transcriptional regulation of polyamine biosynthetic genes, reducing autophagy activity and increasing susceptibility to glomerular injury in inflammatory renal disease models. These cytokines interact with integrin regulatory networks, contributing to disease progression in models of FSGS and lupus nephritis (51). The study used bioinformatics analysis of glomerular expression datasets, alongside immunohistochemical validation in human biopsy samples. Immunohistochemistry and electron microscopy of patient biopsies revealed structural damage to the filtration barrier.

The effect of inflammatory cytokines on FLOT2 regulation in diabetic kidney disease (DKD) was examined in China, where it was found that pro-inflammatory mediators such as tumor necrosis factor-alpha (TNF-α) and interleukin-6 (IL-6) influence FLOT2 transcription via the NF-κB signaling pathway, resulting in disrupted membrane raft stability and altered interactions between FLOT2 and slit diaphragm proteins (77). To assess this, they used cultured podocytes stimulated with cytokines, followed by analyses using Western blotting and immunohistochemistry to quantify FLOT2 expression and examine changes in protein localization.

##### 3.2.2.3 Heavy metals

Exposure to environmental nephrotoxins, including heavy metals (e.g. cadmium and lead), has been associated with podocyte injury and slit diaphragm protein dysregulation, increasing the risk of chronic kidney disease (28). It was reported in the United States that chronic exposure to nephrotoxic heavy metals such as cadmium and lead induces hemolysis and reduces Hp expression, as determined through RT-PCR, Western blotting, and histopathological examination of renal tissues (22). These metals interfere with the CD163 receptor-mediated endocytosis of the ApoHb–Hp complex, resulting in the accumulation of unbound hemoglobin within the glomerular capillaries. The study was performed in murine models, simulating environmental exposure scenarios, and it confirmed that such interference elevates oxidative and inflammatory injury to the kidney.

##### 3.2.2.4 Airborne pollutants

Chronic exposure to fine particulate matter (PM2.5) has been associated with glomerular damage, including podocin dysregulation. In Japan, studies on the impact of air pollution, particularly PM2.5, on podocin among patients with FSGS have been conducted using data from air quality monitoring stations (28).

##### 3.2.2.5 High-fat diet

Diets rich in saturated fats have been associated with podocin downregulation and podocyte stress. Increased lipotoxicity leads to podocyte dysfunction, contributing to obesity-related kidney disease. In Canada, a high-fat diet reportedly contributes to podocin dysfunction in obesity-related kidney disease, using dietary recall methods to assess nutritional intake (78).

##### 3.2.2.6 Vitamins and micronutrient deficiency

Vitamin D has been shown to upregulate podocin expression, protecting against podocyte apoptosis and glomerular injury. Deficiency in vitamin D is linked to increased proteinuria and CKD progression. In Australia, vitamin D deficiency was associated with podocin abnormalities in chronic kidney disease patients, confirmed by serum vitamin D level measurements (79). Vitamin B12 deficiency is common in CKD patients, particularly those on dialysis, and is associated with elevated homocysteine levels, contributing to cardiovascular risk and endothelial dysfunction. Also, vitamin E deficiency increases oxidative stress and inflammation (80). Folate (Vitamin B9) deficiency similarly leads to hyperhomocysteinemia, worsening vascular complications in CKD (80). Deficiency of micronutrients like iron is prevalent in CKD, leading to anemia and reduced oxygen delivery to renal tissues, worsening hypoxia-induced damage (81). Zinc deficiency impairs antioxidant enzyme function (e.g., superoxide dismutase), immune response, and podocyte stability (82).

##### 3.2.2.7 Mechanical stretch

A study investigated how mechanical stretch influences podocyte function (33). Using cultured podocytes subjected to cyclic mechanical stretching to mimic glomerular capillary wall distension during hypertension and hyperfiltration, they found that mechanical stretch induced reorganization of the actin cytoskeleton and increased expression of stretch-sensitive genes, such as transforming growth factor-beta (TGF-β) and connective tissue growth factor (CTGF). These changes contribute to podocyte hypertrophy, detachment, and eventual glomerulosclerosis, highlighting the pathogenic role of mechanical forces in progressive kidney diseases.

##### 3.2.2.8 Nephrotoxic agents

A study investigated the impact of Adriamycin (doxorubicin), a nephrotoxic agent, on podocyte structure and function in a rat model of FSGS (83). The authors found that Adriamycin induced significant podocyte injury characterized by foot process effacement, reduced expression of slit diaphragm proteins such as nephrin and podocin, and cytoskeletal disorganization. These changes led to proteinuria and glomerular scarring. The study demonstrated that nephrotoxic agents directly damage podocytes, contributing to the pathogenesis of glomerular diseases.

## 4 Discussion

### 4.1 Mechanism of slit protein action/pathway

Of all 20 proteins reported in this review, only 15 have been demonstrated in human podocytes and glomerular disease through genetic, immunohistochemical, or biomarker studies. These include key slit-diaphragm scaffolds (nephrin, podocin, CD2AP, MAGI-2), signaling adaptors (PLCE1, Par-complex), actin regulators (synaptopodin, MYO1E), and adhesion proteins (ITGA3, FAT1, podocalyxin, P-cadherin). Ephrin B1, IP-10, Spermidine, Apo Hb–Hp, Neurexin 1, have *emerging or indirect relevance* in humans, with studies mainly in animal or *in vitro* systems. Rather than functioning as isolated components, filtration slit proteins operate within interconnected mechanistic networks like adhesion, cytoskeletal organization, and intracellular signaling. Variations in gene expression or structure, either by inherited mutation or environmental modulation, determine podocyte resilience or vulnerability to injury (42, 44). Reconciling differences across studies reveals that most slit diaphragm defects converge on common downstream pathways, including PI3K–Akt, RhoA/ROCK, NF-κB, and TGF-β/Smad. These pathways integrate genetic susceptibility with oxidative, mechanical, or metabolic stress to determine podocyte fate (49, 50).

#### 4.1.1 Signaling and cytoskeletal regulation

Nephrin (NPHS1) forms the structural and signaling core of the slit diaphragm, linking extracellular filtration forces to actin dynamics. Its tyrosine-phosphorylated intracellular domain recruits Nck and CD2AP, activating PI3K–Akt and Rac1/Cdc42 pathways essential for actin polymerization and podocyte shape (5, 45). Oxidative stress or mutations in *NPHS1* disrupt nephrin phosphorylation, resulting in cytoskeletal collapse and proteinuria (35). Podocin interacts with nephrin at the cytoplasmic side of the slit diaphragm and modulates nephrin’s lipid raft localization. It acts as a scaffold protein that anchors nephrin to the actin cytoskeleton and enhances its signaling through nephrin-PI3K pathways, thus reinforcing slit diaphragm integrity and resisting mechanical stress from filtration pressure (5, 32). Loss of podocin or mutations in *NPHS2* impair raft clustering and attenuate nephrin-mediated PI3K signaling. CD2-associated protein (CD2AP) binds to the intracellular domain of nephrin and links it to the actin cytoskeleton. It acts as a cytoskeletal adaptor, ensuring proper slit diaphragm architecture and facilitating the endocytosis and recycling of membrane components. CD2AP also contributes to the assembly of multi-protein complexes involved in signal transduction and cell survival (46). Together, nephrin, podocin, and CD2AP form a tripartite mechanosensory complex, and their collective disruption results in congenital or steroid-resistant nephrotic syndromes (42).

PLCE1, in contrast, functions as a signaling modulator rather than a structural protein, generating IP_3_ and DAG to activate PKC pathways that regulate cytoskeletal dynamics. Mutations in *PLCE1* cause variable disease phenotypes, indicating that its disruption affects signaling efficiency more than physical architecture (11). Downstream, synaptopodin maintains actin filament plasticity by modulating RhoA and Cdc42 GTPases, while MYO1E acts as a motor protein linking actin to the plasma membrane (14, 48). Loss of these proteins phenocopies the actin instability observed in *NPHS1* or *CD2AP* deficiency, reinforcing the centrality of actin regulation in slit diaphragm maintenance. Genetic variants in *NPHS1*, *NPHS2*, and *CD2AP* demonstrate the strongest causal link to human nephrotic syndromes, reflecting their indispensable structural and signaling functions.

Another important nephrin partner is IQGAP1, which associates with phospholipase Cϵ1 (PLCE1), an enzyme involved in second messenger signaling via inositol 1,4,5-trisphosphate (IP3) and diacylglycerol (84). Mutations in NPHS1 are the primary cause of congenital nephrotic syndrome of the Finnish type, a condition with a high prevalence in Finland (affecting 1 in 10,000 children), as more than 94% of Finnish cases result from two truncating mutations (Fin-major and Fin-minor), reflecting a founder effect (7). Outside Finland, classic NPHS1 mutations are rare, though some missense mutations are associated with a milder FSGS phenotype (85). Similarly, NPHS2 and PLCE1 mutations are linked to steroid-resistant nephrotic syndrome, with clear genotype-phenotype correlations. NPHS2 mutations can cause disease with variable severity and onset ranging from early childhood to early adulthood. PLCE1 truncating mutations generally lead to early-onset proteinuria and rapid progression to kidney failure, whereas missense mutations are linked to later-onset disease with a slower course. Interestingly, some individuals with PLCE1 mutations respond to corticosteroids or immunosuppressive therapy. More recently, mutations in KIRREL1 and KIRREL2, which encode NEPH family proteins that interact with podocin, have been identified in children with steroid-resistant nephrotic syndrome (86, 87).

A common mechanism of podocyte injury in monogenic steroid-resistant nephrotic syndrome appears to be dysregulated calcium signaling. TRPC6, a slit diaphragm–associated calcium channel and mechanical stretch sensor, plays a major role. Gain-of-function mutations in TRPC6 amplify calcium influx, prolonging channel activation and leading to podocyte damage. TRPC6 also mediates angiotensin II–induced calcium influx, contributing to apoptosis and podocyte loss (88). Mutations in other genes, including NPHS2, ACTN4, and APOL1, also result in calcium overload and podocyte injury, likely through increased TRPC6 activity (89).

#### 4.1.2 Adhesion and polarity networks

Podocyte anchorage to the glomerular basement membrane (GBM) is mediated by integrin α3β1 (ITGA3–ITGB1), which connects the extracellular matrix to intracellular actin through focal adhesion complexes (12). Mutations in *ITGA3* compromise adhesion and trigger foot process detachment, leading to nephrotic syndrome and GBM disorganization. FAT1, an atypical cadherin, regulates cell polarity and actin organization via interactions with polarity complexes and β-catenin (44). Both FAT1 and ITGA3 maintain lateral adhesion between neighboring podocytes; their downregulation, as observed in oxidative or mechanical stress, destabilizes the filtration barrier. At the intercellular junction, P-cadherin and neurexin-1 form adherens junctions that couple to the actin cytoskeleton through catenins and MAGI scaffolds. ZO-1 binds directly to nephrin’s cytoplasmic tail, linking it to actin and mediating epithelial polarity. Disruption of these adhesion–polarity networks contributes to podocyte flattening and loss of the filtration slit, phenomena observed in *ITGA3* and *FAT1* knockout models (12, 44).

#### 4.1.3 Membrane raft and redox regulation

Flotillin-2 (FLOT2) stabilizes lipid raft microdomains that host nephrin and podocin. Under oxidative stress, FLOT2 is transiently upregulated to maintain raft integrity; however, chronic exposure to ROS disrupts raft composition, leading to nephrin mislocalization and increased membrane rigidity (10). Podocalyxin is a negatively charged sialomucin expressed on the apical surface of podocytes. It provides electrostatic repulsion between adjacent foot processes, preventing their fusion. Podocalyxin maintains the filtration slit (32). Loss of podocalyxin glycosylation leads to cell fusion and proteinuria. The ApoHemoglobin–Haptoglobin (ApoHb–Hp) complex plays a cytoprotective role by binding free heme and hemoglobin during hemolysis. This interaction prevents oxidative injury and maintains glomerular redox balance (60). In oxidative stress models, Hp deficiency amplifies glomerular ROS accumulation, indirectly promoting nephrin oxidation and actin disassembly.

#### 4.1.4 Metabolic and autophagic modulation

Metabolic homeostasis and autophagic clearance sustain podocyte survival. Spermidine, a polyamine derived from arginine metabolism, enhances autophagy through mTOR inhibition and EP300 acetyltransferase modulation, promoting the clearance of damaged proteins (29). Decreased spermidine synthesis, as seen in oxidative or nutrient stress, leads to autophagic arrest, cytoskeletal disarray, and increased susceptibility to glomerular injury. Liver-type fatty acid–binding protein (L-FABP) mitigates lipid peroxidation and promotes β-oxidation under stress, buffering podocytes against lipotoxicity. High-fat diets suppress L-FABP activity and elevate ROS, thereby amplifying nephrin and podocin downregulation. These mechanisms demonstrate how metabolic stressors intersect with cytoskeletal and membrane signaling pathways to compromise filtration barrier integrity.

### 4.2 Cross-talk between environmental factors and genetic factors to alter the filtration slit protein

The function and integrity of filtration slit proteins are affected by the interaction between genetic programming and environmental exposure. When environmental factors disrupt normal physiology, they often do so by modifying gene expression or protein function through transcriptional, epigenetic, or post-translational mechanisms. This interaction, where environmental signals modify genetic output, is key to understanding the pathophysiology of glomerular diseases.

Oxidative stress induces ROS that interfere with slit diaphragm proteins such as nephrin, podocin, flotillin-2 (Flot2) etc. The ROS activate signaling cascades including MAPK and NF-κB pathways, which in turn suppress NPHS1 and NPHS2 gene transcription (90). In nephrin, oxidative stress increases tyrosine dephosphorylation, leading to its dissociation from CD2AP and actin, resulting in cytoskeletal disorganization and proteinuria (91). Flot2 is upregulated under oxidative conditions, potentially as a compensatory response to maintain raft stability; however, persistent ROS exposure can destabilize these lipid raft domains, promoting mislocalization of Flot2 and altered podocyte morphology.

Also, inflammatory cytokines modulate gene expression by promoting transcription factors such as STAT3 and NF-κB, which downregulate slit protein gene expression or promote pathologic splice variants. For example, TNF-α reduces NPHS1 expression and promotes internalization of nephrin, while also repressing SYNPO gene expression, which impairs synaptopodin’s cytoskeletal stabilization role. IL-6 can reduce FAT1 expression, weakening the lateral adhesion between podocytes and promoting slit diaphragm disruption. Cytokines also stimulate histone deacetylation or DNA methylation in gene promoter regions, silencing genes such as *MYO1E* and CD2AP. Shear stress and stretch activate mechanosensitive signaling pathways such as RhoA/ROCK and YAP/TAZ. These influence the transcription of cytoskeletal and adhesion proteins. For instance, mechanical stress can repress ITGA3 and FAT1 expression while destabilizing podocalyxin through cytoskeletal collapse. This also alters MAGI2 and ZO-1 localization at the junctional complexes, thereby disrupting podocyte polarity and increasing glomerular permeability (82).

Mechanical stress, a physiological consequence of glomerular hypertension or hyperfiltration, activates mechanosensitive pathways such as RhoA/ROCK, YAP/TAZ, and PI3K–Akt. These signaling cascades regulate cytoskeletal gene transcription and junctional protein turnover. Sustained stretch or shear stress downregulates ITGA3 and FAT1, disrupting integrin-mediated adhesion, while destabilizing podocalyxin, MAGI2, and ZO-1 at junctional complexes, leading to loss of polarity and increased glomerular permeability. Furthermore, chronic hyperglycemia, as seen in diabetic nephropathy, drives advanced glycation end-products (AGEs) formation and PKC activation, which alter nephrin and podocin expression through transcriptional repression and post-translational modifications. This includes phosphorylation and ubiquitination, leading to their degradation (90). Hyperglycemia also upregulates FLOT2 and downregulates ITGA3 via ROS and TGF-β1-dependent epigenetic remodeling of their gene promoters, reducing integrin-mediated podocyte adhesion and actin stability.

Diets deficient in antioxidants (e.g., vitamin E, selenium) impair the oxidative defense system, indirectly leading to increased ROS and subsequent gene repression of slit diaphragm proteins. Micronutrient deficiency also affects spermidine synthesis by disrupting polyamine metabolism, resulting in reduced autophagic flux and the accumulation of damaged proteins in podocytes (74). Furthermore, iron deficiency impacts Hp synthesis, reducing the effectiveness of the ApoHb-Hp pathway and increasing oxidative burden on glomeruli. The function and integrity of filtration slit proteins are influenced by the genetic and environmental exposures. Environmental effects alter renal homeostasis by modifying gene expression, protein function, or cellular signaling through transcriptional, epigenetic, or post-translational mechanisms. This complex gene–environment interaction forms the mechanistic basis of glomerular injury and proteinuric diseases.

Heavy metals, such as cadmium and lead, induce epigenetic silencing of nephrin (NPHS1) and podocin (NPHS2) by increasing DNA methylation and histone deacetylation at their gene promoters. This leads to reduced protein expression and impaired slit diaphragm structure (85). Cadmium exposure also disrupts haptoglobin (Hp) and CD163 gene expression, impairing the ApoHb-Hp scavenging pathway, resulting in hemoglobin accumulation, increased oxidative stress, and glomerular toxicity. These agents cause direct podocyte injury and DNA damage, activating p53 and pro-apoptotic genes, while repressing protective genes like SYNPO, PLCE1, and *MYO1E*. Adriamycin nephropathy models have shown upregulation of FLOT2 and decreased expression of NPHS2 and CD2AP, corresponding with foot process effacement and slit diaphragm collapse (87).

Air pollution (e.g., particulate matter, diesel exhaust, polycyclic aromatic hydrocarbons) represents an emerging environmental factor. These pollutants elevate systemic and renal ROS and inflammatory cytokines (TNF-α, IL-6) via AhR and Nrf2 signaling dysregulation. Prolonged exposure leads to oxidative DNA damage, epigenetic silencing of NPHS1 and NPHS2, and mitochondrial dysfunction in podocytes. Particulate matter also increases endothelin-1 and TGF-β1 signaling, inducing podocyte apoptosis and detachment.

### 4.3 Conclusion

This review has mapped out the complex relationship between inherited genetic traits and external environmental factors in modulating the integrity and function of filtration slit proteins, which are essential components of the kidney’s glomerular filtration barrier. One of its key strengths is the systematic approach in synthesizing existing literature, highlighting the connection between genetic predisposition, environmental factors, and disease susceptibility. By discussing specific gene alterations, this review contributes to a deeper understanding of individual variations in kidney disease risk, which may inform personalized therapeutic strategies. Furthermore, the discussion of environmental factors broadens the scope of potential interventions beyond genetic predisposition.

However, certain limitations must be acknowledged. Firstly, while this review integrates findings from multiple studies, variability in study designs, sample sizes, and population demographics may introduce heterogeneity in reported associations. Secondly, the lack of large-scale, multi-ethnic genome-wide association studies limits the generalizability of genetic findings across different populations. As a scoping review that typically maps evidence rather than quality appraisal (8, 92) this study neither conducted a meta-analysis nor did a formal risk-of-bias assessment. Therefore, the presented findings should be understood as a synthesis of evidence, rather than estimates of effect size. Future research should focus on longitudinal studies and randomized controlled trials to validate the role of these proteins in kidney disease progression and therapeutic interventions.

## Data Availability

The original contributions presented in the study are included in the article/**Supplementary Material**. Further inquiries can be directed to the corresponding author.
