# Supplementary material for: Genetic and environmental factors associated with alteration of filtration slit proteins and their functions: a scoping review
Source: Front Nephrol. 2025 Nov 24;5:1678502. doi: 10.3389/fneph.2025.1678502 (PMC12682632; doi:10.3389/fneph.2025.1678502)
Supplement: Supplementary file 1 [file DataSheet1.docx]

**Search strategy for the study titled - Genetic and Environmental Factors Associated with Alteration of Filtration Slit Proteins and Their Functions: A Scoping Review**

**PubMed Search Strategy**

- **Database:** PubMed
- **Date Range:** January 1999 – July 2025
- **Languages:** English
- **Search String:**

(“filtration slit proteins” OR “podocyte proteins” OR “slit diaphragm” OR “nephrin” OR “podocin” OR “CD2AP” OR “FAT1” OR “ITGA3” OR “integrin α3” OR “FLOT2” OR “synaptopodin” OR “actin cytoskeleton” OR “haptoglobin” OR “polyamines”)
AND (“genetic factors” OR “gene mutation” OR “gene regulation” OR “epigenetic” OR “gene polymorphism” OR “knockout mice” OR “loss of function”)
AND (“environmental factors” OR “oxidative stress” OR “hyperglycemia” OR “cytokines” OR “TGF-β” OR “mechanical stress” OR “inflammation” OR “toxin exposure” OR “metabolic stress”)
AND (“glomerular filtration” OR “podocyte injury” OR “proteinuria” OR “focal segmental glomerulosclerosis” OR “nephrotic syndrome” OR “diabetic nephropathy”)

- **Filters Applied:**
  - Species: Humans, Mice, Rats, Zebrafish
  - Study Type: Experimental studies, Clinical studies, Reviews
  - Language: English
- **MeSH Terms Used and Expanded:**
  - *Podocytes* [Mesh]
  - *Nephrin* [Mesh]
  - *Podocin* [Mesh]
  - *Integrins* [Mesh]
  - *Cytoskeleton* [Mesh]
  - *Gene Expression Regulation* [Mesh]
  - *Proteinuria* [Mesh]
  - *Kidney Glomerulus* [Mesh]
  - *Oxidative Stress* [Mesh]
  - *Hyperglycemia* [Mesh]

**Scopus Search Strategy**

- **Database:** Scopus (Elsevier)
- **Date Range:** January 1999 – July 2025
- **Languages:** English
- **Additional Steps:**
  - Combined MeSH terms and free-text keywords using Boolean operators (**AND**, **OR**) to maximize coverage of both molecular and physiological studies.
  - Screened titles and abstracts for relevance to **genetic and environmental modulation of filtration slit proteins** (nephrin, podocin, CD2AP, FAT1, FLOT2, ITGA3, synaptopodin, Par-complex).
  - Manually reviewed reference lists of relevant reviews (e.g., *Ashraf et al., 2020; Pozzi & Zent, 2013; Lennon et al., 2014*) to identify additional primary studies.
  - Included animal model studies (murine, zebrafish) and human genetic investigations where podocyte or filtration slit protein dysfunction was experimentally linked to genetic or environmental stress.
- **Total Records Identified (before screening):** Approximately 650

**Wiley online library**

- **Database:** Wiley online
- **Date Range:** January 1999 – July 2025
- **Languages:** English
- **Search String:**

("filtration slit proteins" OR nephrin OR podocin OR CD2AP OR FAT1 OR FLOT2 OR ITGA3 OR synaptopodin)

AND ("genetic mutation" OR "gene regulation" OR "polymorphism" OR "knockout model")

AND ("oxidative stress" OR hyperglycemia OR inflammation OR "environmental factors")

AND ("podocyte injury" OR "glomerular filtration" OR "proteinuria" OR "nephrotic syndrome")

**Filters:**

- Search restricted to scientific publications, theses, and institutional repositories.
- Focused on articles published in peer-reviewed journals or indexed in PubMed/Scopus.

**Additional Steps:**

- The first 312 results were screened manually.
- Used “Cited by” and “Related articles” features to identify newer or mechanistically related papers.
- Duplicates with PubMed and Scopus entries were removed.
- Search expanded using topic fields (TS) to include title, abstract, and keyword matches.
- Results were screened to include only studies addressing **structural, molecular, or environmental modulation of slit diaphragm proteins**.
